# Tensor Based Semi-Blind Channel Estimation for Reconfigurable Intelligent Surface-Aided Multiple-Input Multiple-Output Communication Systems

**DOI:** 10.3390/s24206625

**Published:** 2024-10-14

**Authors:** Ni Li, Honggui Deng, Fuxin Xu, Yitao Zheng, Mingkang Qu, Wanqing Fu, Nanqing Zhou

**Affiliations:** 1 School of Physics, Central South University, Changsha 410012, China; 222212065@csu.edu.cn (N.L.); xfx.300@163.com (F.X.); 232212089@csu.edu.cn (M.Q.); 2 School of Electronic Information, Central South University, Changsha 410004, China; 222212104@csu.edu.cn (Y.Z.); 232212086@csu.edu.cn (W.F.); zhounanqing@csu.edu.cn (N.Z.)

**Keywords:** reconfigurable intelligent surface, MIMO communication, parallel factor tensor, semi-blind channel estimation, symbol estimation

## Abstract

Reconfigurable intelligent surfaces (RISs) are a promising technology for sixth-generation (6G) wireless networks. However, a fully passive RIS cannot independently process signals. Wireless systems equipped with it often encounter the challenge of large channel matrix dimensions when acquiring channel state information using pilot-assisted algorithms, resulting in high pilot overhead. To address this issue, this article proposes a semi-blind joint channel and symbol estimation receiver without a pilot training stage for RIS-aided multiple-input multiple-output (MIMO) (including massive MIMO) communication systems. In a semi-blind system, a transmission symbol matrix and two channel matrices are coupled within the received signals at the base station (BS). We decouple them by building two parallel factor (PARAFAC) tensor models. Leveraging PARAFAC tensor decomposition, we transform the joint channel and symbol estimation problem into least square (LS) problems, which can be solved by Alternating Least Squares (ALSs). Our proposed scheme allows duplex communication. Compared to recently proposed pilot-based methods and semi-blind receivers, our results demonstrate the superior performance of our proposed algorithm in estimation accuracy and speed.

## 1. Introduction

Reconfigurable Intelligent Surfaces (RISs), an innovative technology in six-generation wireless communication systems, are transitioning from theoretical concepts to practical applications [1,2,3]. Comprising a large array of programmable electromagnetic elements, an RIS could dynamically manipulate the phase, amplitude, and even polarization of incident electromagnetic waves, intelligently shaping and optimizing the wireless signal propagation environment. Its key strengths lie in its non-regenerative nature and low energy consumption, enhancing communication link performance without additional transmission power. Through centralized control or distributed algorithms, RISs adapt in real-time, optimizing beamforming, interference suppression, channel capacity, and coverage, offering unprecedented flexibility and efficiency [4,5,6,7,8].

In a single RIS system, given a fully passive RIS devoid of signal processing capability, the acquisition of Channel State Information (CSI) cannot be directly conducted on the RIS, but necessitates indirect estimation through the base station and user terminals, posing a formidable challenge. Recent works put forward various channel estimation (CE) methods to address the problem [9,10,11,12,13,14,15]. Ref. [9] proposed an eigenspace projection (EP) algorithm based on the correlated nature of RIS–User Terminal (UT) with a pilot reuse strategy to solve the CE problem. Ref. [10] proposed novel long-term and short-term imperfection models, alongside corresponding efficient tensor-based CE algorithms for joint estimation of channels and impairments. In [11], practical broadband CE schemes tailored for multi-path and single-path propagation environments were designed in conjunction with Doppler shift adjustment, effectively addressing the challenges in CE posed by the rapid mobility of users. An efficient alternating optimization algorithm has been proposed in [12], which innovatively integrates sparsity and tensor decomposition structures to formulate the CE problem for RIS-assisted multi-user MIMO systems. In addition to these conventional methods, some researchers focus their attention on deep learning, which can help reduce the CE complexity [16,17,18,19]. A convolutional neural network (CNN)-based denoising network has been proposed in [16] to obtain the CSI. In [17], a time-varying CE scheme based on recurrent neural network (RNN) has been proposed. In [18], the entire channel was estimated by the technique based on the deep back-projection networks (DBPN), which only need small channel samples to train. However, the aforementioned CE methods almost rely on pilot sequence training. The pilot-aided CE approach necessitates the insertion of a predetermined number of pilot symbols within the transmitted signal in a specific arrangement to facilitate CE [20,21]. This entails dedicating a portion of spectral resources to the transmission of known pilot information, rather than actual data, thereby compromising data transmission efficiency and spectral utilization. In addition, in the UT-RIS channel, which is rapidly time-varying, the pilot-aided method may struggle to promptly and accurately track channel variations due to the limited spacing and quantity of pilot signals [22,23]. Consequently, excessively rapid channel changes can render the CE based on pilot signals an inaccurate representation of the current channel state. Furthermore, the extensive deployment of RIS in the system, each equipped with a vast number of elements, leads to a dramatic increase in channel dimensions, thereby resulting in substantial pilot overhead.

To tackle these problems, the semi-blind method has been considered as a potential approach to enhance CE performance and efficiency [24,25,26]. A tensor-based two-stage semi-blind Khatri–Rao factorization and Kronecker factorization (KAKF) receiver was proposed in [24] to jointly estimate channel and transmission symbol matrices without the need for a dedicated pilot training stage. Although the KAKF algorithm without any iterative process shows low computational complexity, it exits error propagation between the two estimation stages, and cannot simultaneously estimate all parameters. Consequently, exploiting the low-rank property of millimetre waves, a tensor-based two-stage semi-blind fitting algorithm was proposed in [25] to preprocess all parameters in the first stage using the Kronecker/Khatri–Rao factorization characteristics. These parameters are then used as initial values for alternate optimization of the channel and symbol matrices in the second stage. The estimation accuracy is improved by adding a preprocessing stage, but it also results in increased computational complexity and a subsequent decrease in estimation speed. Ref. [26] innovatively introduced the PARATUCK tensor model into RIS-assisted MIMO communication systems. Leveraging this model, a semi-blind receiver was designed, which iteratively estimated the channels between the RIS-base station (BS) and UT-RIS, as well as the transmitted symbol matrix, through the trilinear Alternating Least Squares (ALS) method. However, due to the necessity of concurrently optimizing three objective functions, the computational load per iteration becomes exceedingly heavy, resulting in a reduction in the estimation speed.

A semi-blind CE has the advantage of improving spectrum efficiency and lowering pilot overhead; however, how to find a trade-off between the estimation accuracy and speed is still unknown. Motivated by the above, we propose a novel semi-blind receiver based on the ALS algorithm to solve the joint CE and symbol estimation problem. The main contributions of our work are summarized as follows:

- We leverage the superiority in lowering the pilot overhead of the semi-blind CE method. In contrast to conventional pilot-assisted methods, our receiver involves symbol matrix estimation which could lighten the reliance on pilot training.
- We prove that the signal received at the BS adheres to a double parallel factor (PARAFAC) tensor model. The dimension of the received signals can be reduced by leveraging tensor decomposition, resulting in accelerating the estimation speed.
- We propose a novel ALS-based semi-blind receiver for the RIS-aided MIMO communication system. Based on the unfolded forms generated by tensor decomposition, we formulate the joint CE and symbol estimation problem into LS problems and then solve them by ALS.

The remainder of this article is summarized as follows. In Section 2, we give a brief introduction to the parallel factor tensor. The system model is established in Section 3 with joint CE and symbol estimation problem formulation. We present the proposed ALS-based semi-blind technique in Section 4. The simulation results in Section 5 validate the effectiveness of the proposed receiver. We conclude the article in Section 6.

## 2. Preliminaries on the Parallel Factor Tensor Decomposition

PARAFAC decomposition decomposes a high-order tensor into a finite sum of rank-one tensors, providing an approximate representation of the original tensor [27,28,29]. This approach enables dimensionality reduction and information extraction.

**Figure 1:**
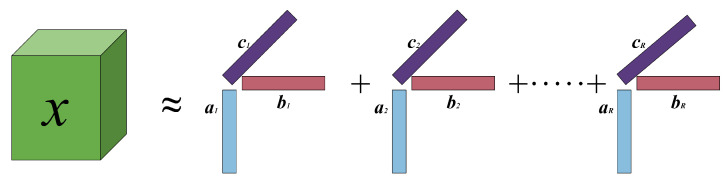
PARAFAC decomposition.

As depicted in Figure 1, for a third-order tensor $X\in C^{I\times J\times K}$, the expression of PARAFAC model can be written as follows:

(1) $$X=I_{R}\times_{1}A\times_{2}B\times_{3}C,$$

where *R* denotes the rank of X, $I_{R}$ denotes the third-order identity tensor of the dimensions R×R×R, the *n*-mode product of a tensor U with a matrix V is denoted by $U\times_{n}V$, the three factor matrices $A=\left[a_{1},\ldots ,a_{R}\right]\in C^{I\times R},B=\left[b_{1},\ldots ,b_{R}\right]\in C^{J\times R}$, and $C=\left[c_{1},\ldots ,c_{R}\right]\in C^{K\times R}$. Then, the PARAFAC decomposition result can be written as

(2) $$\begin{array}{c} X=\sum_{r=1}^{R}{\left[A\right]}_{ir}{\left[B\right]}_{jr}{\left[C\right]}_{kr}\\ \;\mathrm{for}\;i=1,\ldots ,I;j=1,\ldots ,J;k=1,\ldots ,K. \end{array}$$

The decomposition along the three dimensions of the tensor can be represented in matrix form as follows:

(3) $$\begin{array}{c} X\left(i,:,:\right)=B\mathrm{diag}\left(A\right)C^{T}, \end{array}$$

(4) $$\begin{array}{c} X\left(:,j,:\right)=A\mathrm{diag}\left(B\right)C^{T}, \end{array}$$

(5) $$\begin{array}{c} X\left(:,:,k\right)=A\mathrm{diag}\left(C\right)B^{T}. \end{array}$$

To obtain unfolded forms of the mode-1, mode-2, and mode-3, we rewrote (3)–(5) in terms of the horizontal, lateral, and frontal slices of X as

(6) $$\begin{array}{c} \mathrm{Mode-}1:\;{\left[X\right]}_{\left(1\right)}=\left(B⋄C\right)A^{T}, \end{array}$$

(7) $$\begin{array}{c} \mathrm{Mode-}2:\;{\left[X\right]}_{\left(2\right)}=\left(C⋄A\right)B^{T}, \end{array}$$

(8) $$\begin{array}{c} \mathrm{Mode-}3:\;{\left[X\right]}_{\left(3\right)}=\left(A⋄B\right)C^{T}, \end{array}$$

respectively, where ⋄ represents the Khatri–Rao product. Subsequently, we will leverage this PARAFAC decomposition to derive an ALS-based semi-blind receiver.

## 3. System Model

**Figure 2:**
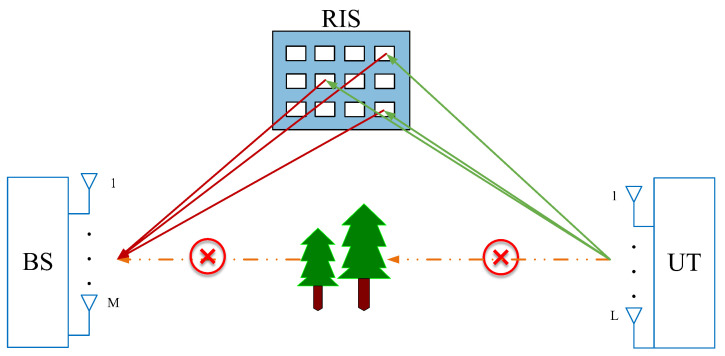
RIS-aided MIMO communication system.

As shown in Figure 2, we consider a fully passive RIS-aided MIMO system along with a quasi-static flat fading channel. To maximize signal coverage, RIS is assumed to be deployed on the surface of a tall building. UT communicates with BS through RIS. The UT-RIS channel varies faster than the RIS-BS channel. The BS and UT are equipped with M and L antennas, respectively. RIS consists of N reflecting elements. Due to an unfavourable propagation environment, our system ignores the direct line-of-sight path between BS and UT.

**Figure 3:**
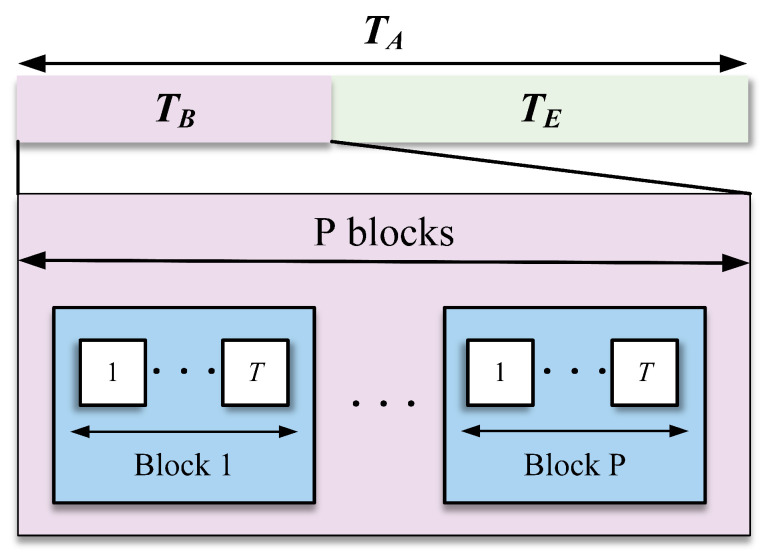
Time protocol.

A transmission frame is taken into account, as depicted in Figure 3. Specifically, coherence time $T_{A}=T_{E}+T_{B}$, $T_{B}$ is significantly smaller than $T_{A}$, where $T_{E}$ and $T_{B}$ represent the data transmission phase and the semi-blind estimation phase, respectively. During the semi-blind estimation duration, $T_{B}$ is divided into P time blocks, and each time block comprises *T* time slots, i.e., $T_{B}=PT\ll T_{A}$.

We focus on an uplink communication scenario, while our proposed scheme is equally applicable to downlink communication. With the assistance of the RIS, the BS receives *L* independent data streams encoded by the UT. The discrete-time signal vectors received by the BS during the *p*-th time blocks (p={1,…,P}) can be expressed as

(9) $$Y_{p,k}=GD_{p}\left(E\right)HD_{k}\left(W\right)X^{T}+B\left[p,k\right],$$

where $G\in C^{M\times N}$ represents the BS-RIS channel, $H\in C^{N\times L}$ represents the RIS-UT channel, $X\in C^{T\times L}$ is the transmitted symbol matrix. Diagonal matrix $D_{i}\left(A\right)$ constructed by placing the i-th row of matrix A along its principal diagonal. The RIS’s phase shift matrix $E\in C^{P\times N}$ is time-varying across blocks, but constant within each block. W∈$C^{K\times L}$ denotes the encoding matrix, where K(k={1,…,K}) is the code length. $B\left[p,k\right]\in C^{M\times T}$ denotes the additive white Gaussian noise matrix. Notations are summarized in Table 1 for convenience. Our objective is to estimate a symbol matrix and two channel matrices; next, we establish two distinct tensor models for the received signals and estimate them in two sequential stages.

## 4. Proposed Semi-Blind Receiver

Observing (9), we find that when *P* or *K* is fixed, the remaining part can be modelled as a three-dimensional tensor that satisfies a PARAFAC decomposition. Hence, we establish two PARAFAC tensor models for the received signals, then estimate them in two separate stages by using ALS fitting.

### 4.1. Symbol Estimation Stage

During the symbol estimation stage, we define F=$GD_{p}\left(E\right)H\in C^{M\times L}$ where F varies across *P* time blocks but remains constant across *K* code length. This implies that F can be fixed when solely considering the received signals that were transmitted using the *k*-th encoding, irrespective of the time block in which they were received. Therefore, we assume that p=1, for simplicity. Then, we rewrite the noise-free received signal in (9) as

(10) $${\tilde{Y}}_{k}=FD_{k}\left(W\right)X^{T}\in C^{M\times T}.$$

The received signal ${\tilde{Y}}_{k}$, which can be regarded as *k*-th frontal matrix slice of tensor, corresponds to the PARAFAC model $\tilde{Y}\in C^{M\times K\times T}$. Unfolding the tensor $\tilde{Y}$ yields the mode-1 and mode-2 forms:

(11) $$\begin{array}{c} \mathrm{Mode-}1:\;{\left[\tilde{Y}\right]}_{\left(1\right)}≜\left(X⋄W\right)F^{T}\in C^{KT\times M}, \end{array}$$

(12) $$\begin{array}{c} \mathrm{Mode-}2:\;{\left[\tilde{Y}\right]}_{\left(2\right)}≜\left(W⋄F\right)X^{T}\in C^{KM\times T}. \end{array}$$

(13) $$\begin{array}{c} \mathrm{Mode-}3:\;{\left[\tilde{Y}\right]}_{\left(3\right)}≜\left(F⋄X\right)W^{T}\in C^{MT\times K}. \end{array}$$

In the (i)-th iteration, utilizing unfolded forms (11) and (12), we perform Least Squares (LS) fitting to obtain an estimate of X and F by minimizing the following cost functions:

(14) $$\begin{array}{c} J\left({\hat{X}}^{i}\right)={∥{\left[\tilde{Y}\right]}_{\left(2\right)}-\left(W⋄{\hat{F}}^{i-1}\right){\left({\hat{X}}^{i-1}\right)}^{T}∥}_{F}^{2}, \end{array}$$

(15) $$\begin{array}{c} J\left({\hat{F}}^{i}\right)={∥{\left[\tilde{Y}\right]}_{\left(2\right)}-\left({\hat{X}}^{i}⋄W\right){\left({\hat{F}}^{i}\right)}^{T}∥}_{F}^{2}, \end{array}$$

and the closed-form solutions are given as follows:

(16) $$\begin{array}{c} {\hat{X}}^{i}={\left({\left(W⋄{\hat{F}}^{i-1}\right)}^{†}{\left[\tilde{Y}\right]}_{\left(2\right)}\right)}^{T}, \end{array}$$

(17) $$\begin{array}{c} {\hat{F}}^{i}={\left({\left({\hat{X}}^{i}⋄W\right)}^{†}{\left[\tilde{Y}\right]}_{\left(1\right)}\right)}^{T}. \end{array}$$

where ${\left(\cdot \right)}^{†}$ represents the pseudo-inverse of the matrix.

### 4.2. Channel Estimation Stage

Since we acquire the estimated values of X from the stage I and the encoding matrix W is known in BS, we can reconstruct the received signal by removing X and W. Specifically, we can perform the following operation:

(18) $$\begin{array}{cc} {\tilde{Y}}_{p}= & GD_{p}\left(E\right)HD_{k}\left(W\right)X^{T}{\left({\hat{X}}^{T}\right)}^{†}D_{k}{\left(W\right)}^{-1}\\ \; & +B\left[p,k\right]{\left({\hat{X}}^{T}\right)}^{†}D_{k}{\left(W\right)}^{-1}. \end{array}$$

Let $Q={\left[HD_{k}\left(W\right)X^{T}{\left({\hat{X}}^{T}\right)}^{†}D_{k}{\left(W\right)}^{-1}\right]}^{T}\in C^{L\times N}$, where Q varies across *K* code length but remains constant across *P* time blocks. Since we only consider the received signal received in *p*-th time block regardless of which code is employed for their transmission, the variable Q is fixed by assuming k=1 for simplicity. Then, we rewrite the noise-free received signal in (18):

(19) $${\tilde{Z}}_{p}=GD_{p}\left(E\right)Q^{T}\in C^{M\times L}.$$

The ${\tilde{Z}}_{p}$ can be regarded as *p*-th frontal matrix slice of tensor. Based on (19), we establish a tensor $\tilde{Z}\in C^{M\times P\times L}$ and apply PARAFAC decomposition, then obtain unfolded forms of the mode-1 and mode-2 of $\tilde{Z}$:

(20) $$\begin{array}{c} \mathrm{Mode-}1:\;{\left[\tilde{Z}\right]}_{\left(1\right)}≜\left(Q⋄E\right)G^{T}\in C^{PL\times M}, \end{array}$$

(21) $$\begin{array}{c} \mathrm{Mode-}2:\;{\left[\tilde{Z}\right]}_{\left(2\right)}≜\left(E⋄G\right)Q^{T}\in C^{PM\times L}. \end{array}$$

(22) $$\begin{array}{c} \mathrm{Mode-}3:\;{\left[\tilde{Z}\right]}_{\left(3\right)}≜\left(G⋄Q\right)E^{T}\in C^{ML\times P}. \end{array}$$

During the (i)-th iteration, to estimate Q and G, we employ the same ALS fitting as in stage I. Leveraging the received signal unfolded forms (20) and (21), the cost functions are given by

(23) $$\begin{array}{c} J\left({\hat{Q}}^{i}\right)={∥{\left[\tilde{Z}\right]}_{\left(2\right)}-\left(E⋄{\hat{G}}^{i-1}\right){\left({\hat{Q}}^{i-1}\right)}^{T}∥}_{F}^{2}, \end{array}$$

(24) $$\begin{array}{c} J\left({\hat{G}}^{i}\right)={∥{\left[\tilde{Z}\right]}_{\left(1\right)}-\left({\hat{Q}}^{i}⋄E\right){\left({\hat{G}}^{i}\right)}^{T}∥}_{F}^{2}, \end{array}$$

Then, the closed-form solutions of Q and G can be derived as

(25) $$\begin{array}{c} {\hat{Q}}^{i}={\left({\left(E⋄{\hat{G}}^{i-1}\right)}^{†}{\left[\tilde{Z}\right]}_{\left(2\right)}\right)}^{T}, \end{array}$$

(26) $$\begin{array}{c} {\hat{G}}^{i}={\left({\left({\hat{Q}}^{i}⋄E\right)}^{†}{\left[\tilde{Z}\right]}_{\left(1\right)}\right)}^{T}. \end{array}$$

The normalized squared error at the (i)-th iteration for the two stages are

(27) $$\begin{array}{c} e_{\left(i\right)}^{\alpha }={∥{\left[\tilde{Y}\right]}_{\left(1\right)}-\left({\hat{X}}^{i}⋄W\right){{\hat{F}}^{i}}^{T}∥}_{F}^{2}, \end{array}$$

(28) $$\begin{array}{c} e_{\left(i\right)}^{\beta }={∥{\left[\tilde{Z}\right]}_{\left(1\right)}-\left({\hat{Q}}^{i}⋄E\right){{\hat{G}}^{i}}^{T}∥}_{F}^{2}. \end{array}$$

We set the iteration terminating criterion for both two stages as $\left|e_{\left(i\right)}^{\chi }-e_{\left(i-1\right)}^{\chi }\right|\leq \varepsilon$, where χ=α,β and the threshold value $\varepsilon =10^{-6}$. Actually, the estimation result of channel H equal to the estimated Q, i.e., $\hat{H}=\hat{Q}$. The proposed double PARAFAC-based ALS (DP-ALS) CE is summarized in Algorithm 1.
**Algorithm 1:** Proposed double-PARAFAC-based Alternating Least Squares.**Input:** Initialize $F^{\left(1\right)},G^{\left(1\right)}$. 1: Symbol Estimation Stage 1.1: i=1; 1.2: **While** $|e_{\left(i\right)}^{\alpha }-e_{\left(i-1\right)}^{\alpha }|>\varepsilon$ **do** 1.3:      i=i+1; 1.4:      Calculate ${\hat{X}}^{i}$ by (16); 1.5:      Calculate ${\hat{F}}^{i}$ by (17); 1.6: **End While** 2: Channel Estimation Stage 2.1: i=1; Reconstruct receive signals by (18); 2.2: **While** $|e_{\left(i\right)}^{\beta }-e_{\left(i-1\right)}^{\beta }|>\varepsilon$ **do** 2.3:      i=i+1; 2.4:      Calculate ${\hat{Q}}^{i}$ by (25); 2.5:      Calculate ${\hat{G}}^{i}$ by (26); 2.6: **End While** Output: $\hat{X},\hat{G},\hat{Q}$.


### 4.3. Computational Complexity

The computational complexity of the proposed algorithms mainly lies in the matrix pseudo-inverse operations, which are contained in steps 1.4, 1.5, 2.1, 2.4, 2.5 of Algorithm 1 with complexity orders of $O\left(L^{3}\right)$, $O\left(L^{3}\right)$, $O\left(L^{3}\right)$, $O\left(N^{3}\right)$, and $O\left(N^{3}\right)$, respectively. Therefore, the total complexity of Algorithm 1 is $O\left(3L^{3}+2N^{3}\right)$, respectively. The dominant complexity of the PARAFAC-based pilot-assisted receiver [13], KAKF semi-blind receiver [24], and TALS semi-blind receiver [26] are $O\left(\left(M+L\right)KN^{2}\right)$, $O\left(MT+TLN^{2}M^{2}\right)$, and $O(TKN^{2}$ + $LKM^{2})$, respectively. Our proposed algorithm has lower computational complexity compared to these competitive algorithms. Moreover, to eliminate the scale ambiguity inherent in Algorithm 1, we normalize the first column of X and H. It is assumed that the first column of symbol X and UT-RIS channel H are known in BS.

### 4.4. Feasibility Analysis

To ensure unique solutions for G,Q, and X, it is necessary to guarantee that all pseudo-inverses within the algorithm are valid, i.e., $X⋄W\in C^{KT\times L},W⋄F\in C^{KM\times L},Q⋄E\in C^{PL\times N}$, and $E⋄G\in C^{PM\times N}$ must have full column rank. Specifically,

(29) $$\left\{\begin{array}{c} K\min(T,M)\geq L,\\ P\min(L,M)\geq N. \end{array}\right.$$

In addition, the uniqueness conditions for the PARAFAC decomposition must also be met. According to the identifiability theorem of the PARAFAC tensor model, it can be proven that, in the proposed receiver, if

(30) $$\begin{array}{c} k_{F}+k_{X}+k_{W}\geq 2L+2, \end{array}$$

(31) $$\begin{array}{c} k_{G}+k_{Q}+k_{E}\geq 2N+2, \end{array}$$

where $k_{A}$ denote the Kruskal rank of A, then (F,X,W) and (G,Q,E) possess uniqueness in terms of permutation and scaling. Consequently, there exist corresponding permutation matrices and diagonal scaling matrices for (F,X,W) and (G,Q,E), which can restore the original estimates of the matrices, i.e.,

(32) $$\begin{array}{cc} \; & \hat{F}=F\Pi_{1}\Delta_{1}^{1},\hat{W}=W\Pi_{1}\Delta_{2}^{1},\hat{X}=X\Pi_{1}\Delta_{3}^{1}, \end{array}$$

(33) $$\begin{array}{cc} \; & \hat{G}=G\Pi_{2}\Delta_{1}^{2},\hat{E}=E\Pi_{2}\Delta_{2}^{2},\hat{Q}=Q\Pi_{2}\Delta_{3}^{2}, \end{array}$$

where $\Pi_{1}$ and $\Pi_{1}$ are permutation matrices of size L×L and N×N, respectively, and $\Delta_{1,2,3}^{1}$ and $\Delta_{1,2,3}^{2}$ are diagonal scaling matrices of size L×L and N×N, respectively, with the conditions $\Delta_{1}^{1}\Delta_{2}^{1}\Delta_{3}^{1}=I_{L}$ and $\Delta_{1}^{2}\Delta_{2}^{2}\Delta_{3}^{2}=I_{N}$. Given that the channel model and the symbol matrix considered in this letter, X,G, and Q are full-rank matrices, Conditions (29) and (30) can be translated into

(34) $$\begin{array}{c} \min\left(M,L\right)+\min\left(T,L\right)+\min\left(K,L\right)\geq 2L+2, \end{array}$$

(35) $$\begin{array}{c} \min\left(M,N\right)+\min\left(L,N\right)+\min\left(P,N\right)\geq 2N+2. \end{array}$$

Therefore, system parameters should satisfy min{M,T}≥L during stage I, and min{M,L}≥N during stage II. Furthermore, to flexibly adapt to varying environmental communication requirements, the number of RIS-reflecting elements may exceed BS or UT antennas. In such cases, if the feasibility conditions are not met, we can partition the RIS-reflecting elements into non-overlapping subgroups, employing the proposed algorithms to estimate the channels of each subgroup. Subsequently, the estimated results can be combined to obtain the desired CSI [13].

## 5. Simulations and Results

In this section, based on the simulation results, we give an evaluation of our proposed algorithm in terms of normalized mean square error (NMSE), symbol error rate (SER) and average run time, as well as the iterations to converge. The NMSE for CE is calculated using

(36) $$\mathrm{NMSE}\left(\hat{\Theta }\right)=\frac{1}{j}\sum_{j=1}^{J}\frac{{∥\Theta^{\left(j\right)}-{\hat{\Theta }}^{\left(j\right)}∥}_{F}^{2}}{{∥\Theta^{\left(j\right)}∥}_{F}^{2}},$$

where Θ={H,G} and ${\hat{\Theta }}^{\left(j\right)}$ denote the estimated value at the *j*-th iteration. The index *j* refers to the Monte Carlo simulation iteration. All simulation results are averaged over 6000 independent Monte Carlo runs. For the channel model, we consider a Rayleigh fading channel, where G and H have independent and identically distributed complex Gaussian entries. The transmitted data symbols are drawn from a 16PSK alphabet. We chose the first *P* and *K* rows of N×N and L×L DFT matrices, respectively, to form W and E. The simulation parameters are set as follows: M=5,L=2,T=5,N=32,K=32, P=128.

The comprehensive comparative analysis is conducted against recently proposed receivers, including the PARAFAC-based pilot-assisted receiver [13], KAKF semi-blind receiver [24], and TALS semi-blind receiver [26]. Notably, these receivers employ diverse methodologies to tackle the objective function: the PARAFAC-based pilot-assisted and TALS semi-blind receivers utilize iterative solutions involving two/three Least Squares problems for estimating channel or transmission symbol matrix. In contrast, the KAKF semi-blind receiver adopts a closed-form solution based on rank-one matrix approximation without iteration stage.

**Figure 4:**
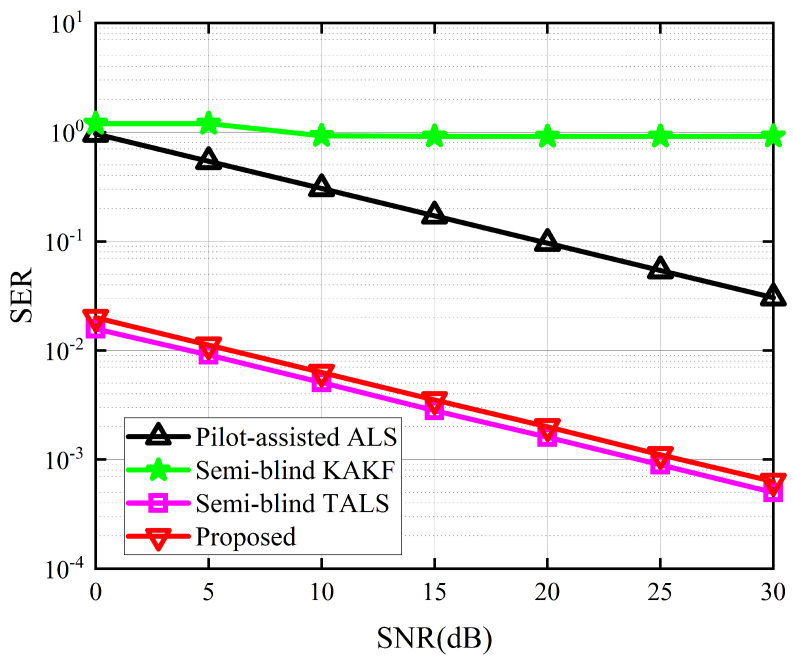
SER between the different receivers.

**Figure 5:**
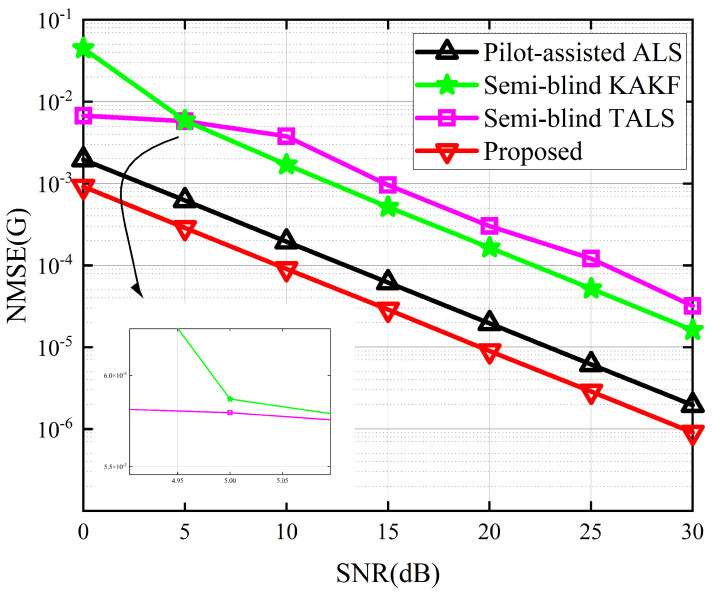
NMSE for the channel G between the different receivers.

**Figure 6:**
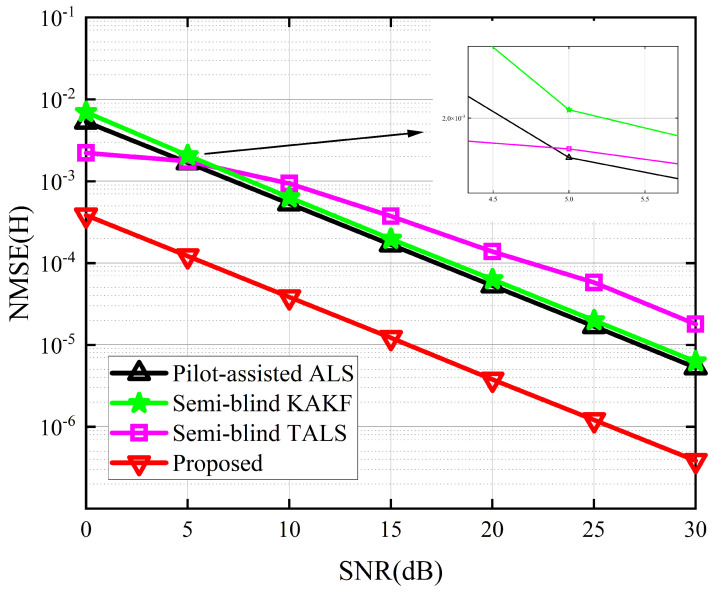
NMSE for the channel H between the different receivers.

The performance of the proposed receiver in terms of SER and NMSE of G and H are depicted in Figure 4, Figure 5 and Figure 6. Due to the absence of an iterative process in the symbol matrix estimation phase of the KAKF receiver, its SER remains invariant with varying signal-to-noise ratio (SNR). Figure 4 shows that the proposed receiver outperforms the ALS receiver and the KAKF receiver. Specifically, compared to the worst-performing KAKF receiver, the proposed algorithm exhibits a reduction in SER by two to three orders of magnitude. This is attributed to the structural advantage of the ALS algorithm, which involves iteratively refining the feature matrix in a step-by-step manner, with each iteration contributing to a reduction in error. The proposed receiver lags slightly behind the TALS receiver by approximately a 2 dB SNR gap. This is owing to the TALS receiver estimates the matrices simultaneously. In contrast, our proposed receiver is divided into two phases to estimate them, resulting in the parameters of our proposed receiver engaged in the symbol estimation stage being less than those of the TALS receiver. However, it is acceptable to sacrifice a small portion of SER performance to achieve greater gains in NMSE. From Figure 5 and Figure 6, we can see that the proposed receiver has gained more accurate channel estimation in comparison with other methods. Specifically, the proposed receiver exhibited a minimal SNR gap of approximately 3 dB and 16 dB for G channel and H channel, respectively. This is because our proposed method contains two tensor decomposition processes, which could be regarded as denoise operations. The superiority in CE of our proposed algorithm also implies that, even though errors would propagate between the two estimation stages in the proposed receiver, we address this by reconstructing the received signals in the second stage and treating the propagation errors as noise for CE. Then, we exploit the noise reduction property of PARAFAC decomposition, thereby effectively mitigating the propagation errors.

**Figure 7:**
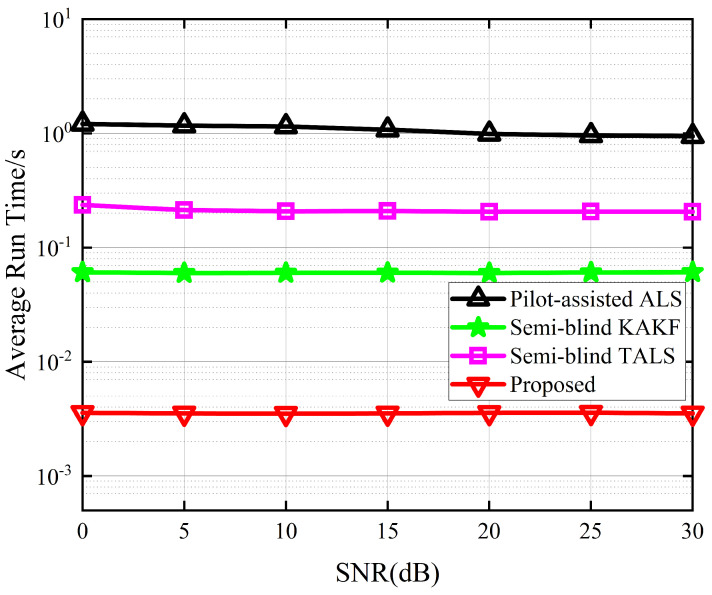
Average run time between the different receivers.

**Figure 8:**
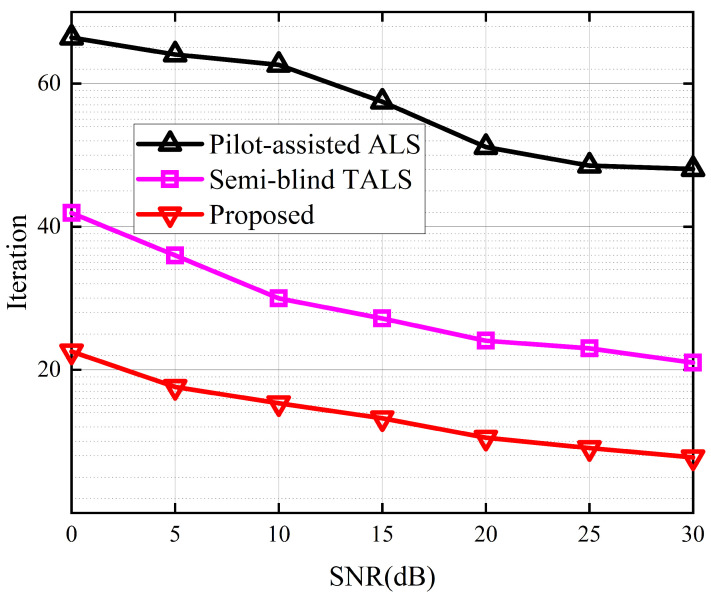
Iterations to converge between the different receivers.

Figure 7 depicts the average run time between the different receivers. The average run time takes into account the number of iterations required for convergence, thereby enabling a comparison of the complexity between iterative and closed-form solutions. The results show that the proposed receiver outperforms the other three receivers. In comparison to the best-performing KAKF receiver, the proposed algorithm demonstrates a decrease in average run time by almost one order of magnitude. This is attributed to the exploitation of the low-rank property of tensor decomposition, which enables us to reduce the dimensionality of the received signal. Figure 8 depicts the iterations to converge between the different receivers. From Figure 8, we can see that the proposed receiver achieved a minimum number of convergence iterations. This is because we harness the inherent mathematical properties embedded within the received signals to simplify the objective function, thereby facilitating the convergence of the cost function during the alternating optimization process for the symbol matrix and the channel matrices.

## 6. Conclusions

For single-RIS-aided MIMO communication scenarios, we have proposed novel semi-blind receivers to address the challenge of accurately and rapidly acquiring channel state information. We carefully decoupled the signal reflected by the RIS and received at the BS into two signals that both conformed to a generalized PARAFAC tensor model. Then, we turn the joint CE and symbol estimation problem into four LS subproblems through PARAFAC decomposition. The UT-RIS and RIS-BS channels and symbols could obtained by performing the ALS algorithm. In addition, we investigated the feasibility conditions and complexity of the proposed receiver. The proposed receiver enables the joint estimation of UT-RIS and RIS-BS channels, as well as transmitted symbols, without the need for dedicated pilot training. Compared to recent semi-blind receivers, the proposed receiver exhibits less computational complexity, fewer iterations to converge, and superior performance in channels’ NMSE. Simulation results demonstrate those superior performances of our receiver achieved.

## Figures and Tables

**Table 1:** Summary of notations.

Notation	Meaning
M	The number of BS antennas
L	The number of UT antennas
N	The number of reflecting elements
P	The number of time blocks
T	The number of time slots
K	Code length
G	BS-RIS channel
H	RIS-UT channel
X	Transmitted symbol matrix
E	RIS’s phase shift matrix
W	Encoding matrix
B	Additive white Gaussian noise matrix

## Data Availability

Data are contained within the article.
